# Application of In-Home Monitoring Data to Transition Decisions in Continuing Care Retirement Communities: Usability Study

**DOI:** 10.2196/18806

**Published:** 2021-01-13

**Authors:** Katherine Wild, Nicole Sharma, Nora Mattek, Jason Karlawish, Thomas Riley, Jeffrey Kaye

**Affiliations:** 1 Department of Neurology Oregon Center for Aging and Technology Oregon Health and Science University Portland, OR United States; 2 Department of Medicine University of Pennsylvania Philadelphia, PA United States; 3 Department of Medical Ethics and Health Policy University of Pennsylvania Philadelphia, PA United States; 4 Department of Neurology University of Pennsylvania Philadelphia, PA United States; 5 Department of Biomedical Engineering Oregon Center for Aging and Technology Oregon Health and Science University Portland, OR United States

**Keywords:** technology, remote sensing technology, care transition

## Abstract

**Background:**

Continuous in-home monitoring of older adults can provide rich and sensitive data capturing subtle behavioral and cognitive changes. Our previous work has identified multiple metrics that describe meaningful trends in daily activities over time. The continuous, multidomain nature of this technology may also serve to inform caregivers of the need for higher levels of care to maintain the health and safety of at-risk older adults. Accordingly, care decisions can be based on objective, systematically assessed real-time data.

**Objective:**

This study deployed a suite of in-home monitoring technologies to detect changing levels of care needs in residents of independent living units in 7 retirement communities and to assess the efficacy of computer-based tools in informing decisions regarding care transitions.

**Methods:**

Continuous activity data were presented via an interactive, web-based tool to the staff identified in each facility who were involved in decisions regarding transitions in care among residents. Comparisons were planned between outcomes for residents whose data were shared and those whose data were not made available to the staff. Staff use of the data dashboard was monitored throughout the study, and exit interviews with the staff were conducted to explicate staff interaction with the data platform. Residents were sent weekly self-report questionnaires to document any health- or care-related changes.

**Results:**

During the study period, 30 of the 95 residents (32%) reported at least one incidence of new or increased provision of care; 6 residents made a permanent move to a higher level of care within their communities. Despite initial enthusiasm and an iterative process of refinement of measures and modes of data presentation based on staff input, actual inspection and therefore the use of resident data were well below expectation. In total, 11 of the 25 staff participants (44%) logged in to the activity dashboard throughout the study. Survey data and in-depth interviews provided insight into the mismatch between intended and actual use.

**Conclusions:**

Most continuous in-home monitoring technology acceptance models focus on perceived usefulness and ease of use and equate the intent to use technology with actual use. Our experience suggests otherwise. We found that multiple intervening variables exist between perceived usefulness, intent to use, and actual use. Ethical, institutional, and social factors are considered in their roles as determinants of use.

## Introduction

Although factors associated with older adults’ moves to residential facilities have been well described [1-4], our understanding of the reasons for changes in the level of care in persons who live in continuing care retirement communities (CCRCs) is limited. However, there appears to be some overlap with transitions from independent community living. Increased confusion, loss of mobility, medication nonadherence, and reduced socialization have been identified as predictors of movement to higher levels of care in a CCRC [5-8].

A delay in identification of increased dependence or inability to perform routine self-care activities can result in costly and potentially dangerous outcomes for at-risk residents. Typically, decisions about care needs and transitions in the levels of care for older adults living in CCRCs rely on communication and coordination among professional staff members. Ideally, these decisions are based on the evaluations of relevant health and behavioral changes. As Couture et al [9] have argued, the decision-making process is best accomplished by input from all stakeholders across health care professions as well as from family members and the residents themselves. Georgiou et al [10] identified barriers to optimal communication in residential care facilities, which may have a negative impact on the provision of quality care related to efficient and timely transitions to different levels of care. Another challenge to judicious care transitions is the relative inability to detect changes in a resident’s care needs before an acute event occurs.

It has been noted that policies and procedures may not be suitable for efficient and timely information transfer. Kelsey et al [5] reported that policies for transfer from one level of care to another vary across facilities, ranging from a multidisciplinary team approach to decision making by a facility manager or administrator. They recommend that future research attention be paid to the ultimate appropriateness of resident transfers to higher levels of care. In the long term, codification of procedures and identification of factors contributing to moves within a residential community can serve to reduce resistance and misunderstanding and may have the potential to enhance safety and prolong independence in at-risk older adults.

A lack of formalized protocols or objective behavioral markers to guide the process may contribute to the uncertainty and divergent views associated with the determination of residents’ needs for transition from independent to more assisted living within a continuing care setting. One way to compile objective, systematically assessed activity data is through continuous monitoring technologies. Demiris and Thompson [11] have cited the value of in-home activity monitoring technologies in delivering large, individually anchored data sets that are *useful, meaningful, and actionable*.

Systems that improve our ability to unobtrusively monitor important health changes because of chronic disease and aging could allow timely intervention that prevents avoidable loss of independence. Continuous collection of health and activity information in the home can enable early identification of clinically significant changes. Our experience in examining the feasibility of deploying a comprehensive, ubiquitous sensing platform in the homes of older adults has been reported previously [12,13]. ORCATECH (Oregon Center for Aging & Technology) research has demonstrated the sensitivity of unobtrusive in-home technology to detect early changes in medication management capacity [14,15], patterns of mobility [16-18], nighttime sleep behaviors [19], computer use [20,21], and driving [22]. Such technologies can provide important information in guiding decisions regarding increased care needs. We aimed to test the hypothesis that providing objective and continuous data from home-based technologies to the care teams in retirement communities will result in fewer transitions to higher levels of care through early identification of behavioral or activity changes that lead to increased in-home assistance.

In this paper, we report on the results of Ambient Independence Measures for Guiding Care Transitions (AIMS), a study that provided designated staff at 7 continuing care residential communities with access to an automated, continuous data monitoring platform via a web-based dashboard that collected residents’ behavioral and physiological sensor-based independence metrics. Owing to low use of data by the staff, the trial was not able to adequately evaluate the primary hypothesis that providing these data will result in fewer transitions to higher levels of care and increase in-home assistance because of early identification of potential problems. We describe the procedures implemented to maintain staff engagement and consider the challenges of new technology adoption in residential facilities. Using exit interviews with the staff, we examined the reasons for the low use of the data to recommend how studies of home monitoring of health and activity can be improved.

## Methods

### Overview

Before initiating the trial, focus group sessions were conducted with care transition teams at the participating retirement communities to better understand their routine process of making decisions about when residents need to transition to a higher level of care and to receive feedback on the proposed AIMS data provision interface. Their recommendations were incorporated into the final dashboard interface where feasible. Residents were then monitored for 3 years, with behavior and activity data supplied to identified staff for half the study residents.

### Recruitment

#### Older Adult Participants

Individuals were recruited from existing registries of volunteers for research residing in one of 7 retirement communities in the Portland metropolitan area and during presentations given at these communities about the project. These residential facilities participated in previous and ongoing research about technology and aging. Demographic data were collected at the time of enrollment in this study or other ORCATECH studies. Participants were aged 75 years or older, independently living in an apartment that was larger than one room, living alone, not demented, and of average health for their age (ie, without a medical illness that would limit physical participation or possibly lead to death over the next 36 months). As part of their participation in this study, the residents agreed to have their in-home activity data shared with their facility staff and were required to have internet service and to be computer users. The cohort was allocated to having the staff view their in-home activity via the web-based dashboard versus no viewing, using simple randomization (computer-generated assignment) at a 1:1 ratio, with an enrollment goal of 50 residents per group. The observation period for the study was 3 years.

Resident participants were instructed to live their lives without any specific health or activity intervention. They replied once a week to an email that directly queried them regarding health or activity change (mood, pain, loneliness, falls, hospital visits, visitors, and limited activity due to health) as well as the need for new or additional care.

#### Staff Participants

A total of 25 staff members at these facilities were self-identified as participants in decision making around residents’ transitions in levels of care. They were recruited to this study during regularly scheduled staff meetings. Staff were aged between 21 and 66 years (mean 39.6, SD 10.4 years) and were employed at their present facility for an average of 10 years (range 2 weeks to 16 years). Staff levels of education ranged from high school graduate to master’s degree; a majority (17/25, 68%) had obtained at least a college degree. Their job titles varied and included directors, nurses, social workers, and resident care coordinators.

### Monitoring Platform

The details of the home technology system have been described previously [12,13]. In brief, sensors and other in-home technologies are deployed to continuously monitor daily activities. In response to specific queries from the research team regarding the dashboard content and interface, the staff made multiple concrete suggestions on all aspects, ranging from how to better navigate the site to changing the symbols used to represent alerts for changes in activity status. On the basis of the input from the care teams, a study protocol was finalized, which focused on home-based sensors and devices whose data were perceived as indicating that major functions had changed and thus could influence independent living decisions. The selected metrics included mobility, physiological health, nighttime behaviors, medication adherence, socialization, cognitive function, and self-reported health changes via a web-based weekly health form (Table 1).

**Table 1:** Ambient Independence Measures for Guiding Care Transitions study metrics and devices.

Core functions	Measures	Sensors or devices used
Physical capacity and mobility	Total daily activity, number of room transitions, typing speed, time out of home	PIR^a^ motion sensors and contact sensors, computer use metrics (eg, keyboard trigrams)
Walking speed	Median weekly walking speed from multiple daily walks	PIR motion sensor line
Sleep and nighttime behavior	Time of awakening in the morning, time spent in bed at night, wake after sleep onset, times up at night, sleep latency	PIR motion sensors
Physiologic health	Daily BMI, morning pulse	Bioimpedance scale
Medication adherence	Percentage of doses missed in a 7-day period relative to prescribed schedule	MedTracker electronic pillbox
Socialization and engagement	Time out of home, time alone, phone call patterns, online computer activity (email and social networking sites)	PIR motion sensors, contact sensors, personal computer, phone monitors
Cognitive function	Time to complete online tasks, mouse movements, prospective memory for medication	Personal computer or tablet, MedTracker
Health and life events	Online self-report: emergency room, doctor, hospital visits, home visitors, mood, pain, loneliness, falls, injuries, change in home space, home assistance received, change in medications	Personal computer or tablet

^a^PIR: passive infrared.

**Figure 1:**
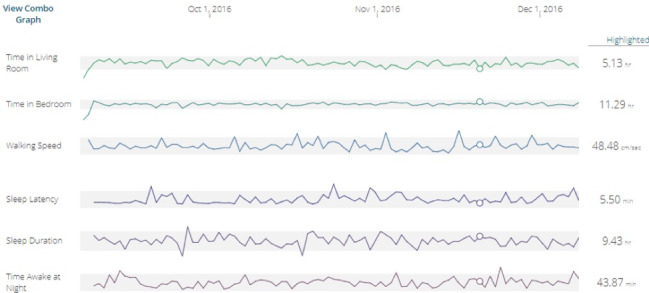
Sample dashboard display (screenshot) of continuously assessed in-home activity metrics in Ambient Independence Measures for Guiding Care Transitions (AIMS) residents. The display, showing data at the individual level, can be customized by the user to show higher-level summaries, single metrics, numerical detail, and different windows of time. In this custom view, multiple metrics are displayed (eg, time out of home, physiologic measures, sleep measures, bathroom trips). The gray shading indicates preset ranges.

**Figure 2:**
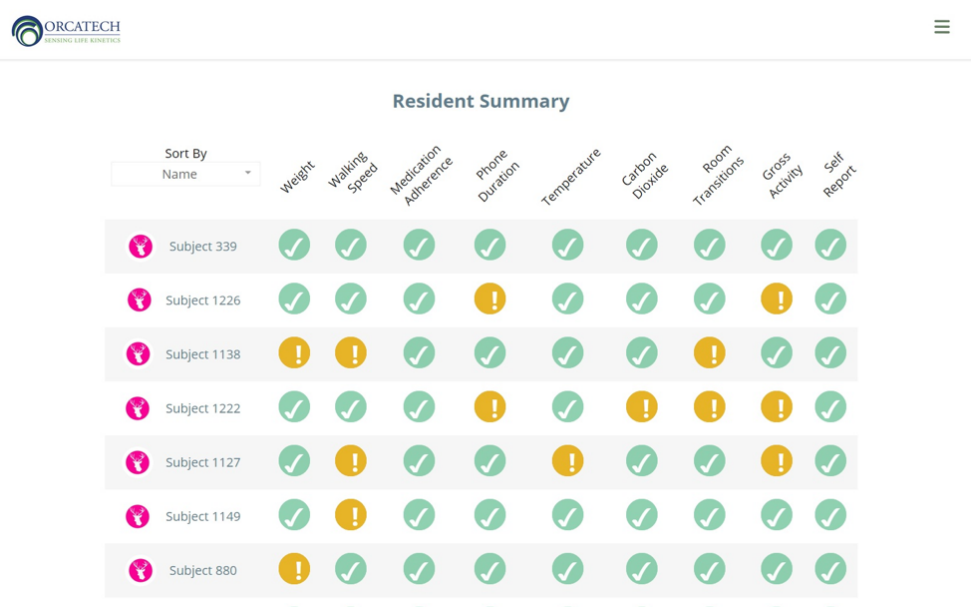
Sample dashboard display of residents’ summary data. Exclamation points indicate departure from the usual level of behavior or activity for each resident (Temperature and Carbon Dioxide refer to environmental metrics and were not included in this study).

Resident homes were installed with a sensor platform consisting of passive infrared motion and contact sensors, MedTrackers, weight scales, and software to capture all metrics as well as residents’ computer use. A web-based reporting tool was developed to track these data, identify outlying data and trends, and provide staff with access to views of these data on a variety of timescales in a dashboard format that was available to the participating facilities’ care transition teams. Figure 1 presents an example of a dashboard data summary for a single resident across various metrics over time. The dashboard interface was designed to be interactive, allowing for the manipulation of time scale, activity, and residents of interest. It also provided alerts to the staff for behaviors and activities that fell outside the range of normal for any particular resident. The basic approach for alerts was to develop a baseline model of typical measures for each individual over multiple weeks and monitor these measures on a regular basis (eg, weekly) for trends away from the norm. Alerts were embedded in data summaries for individual behaviors and activities. Figure 2 depicts a sample resident’s summary graphic, where outlying behaviors are represented by an alert (!) and green checkmarks indicate the usual behavior *for that resident*.

### Staff Training and Engagement

Facility staff who had been identified as part of the transition assessment team and were willing to participate in the study were scheduled for 1-hour training sessions by AIMS study staff. Each staff member also received a printed dashboard user guide with detailed instructions on the use of the dashboard and its functionalities in terms of resident activity categories, data summaries, and useful comparisons across activities and time frames. Contact information for additional help was included, and technicians returned to sites to provide additional training as needed. During the study period, minimal additional training was requested; our technician made 2 additional visits to facilities and provided occasional help by telephone. These data are not tabulated.

**Figure 3:**
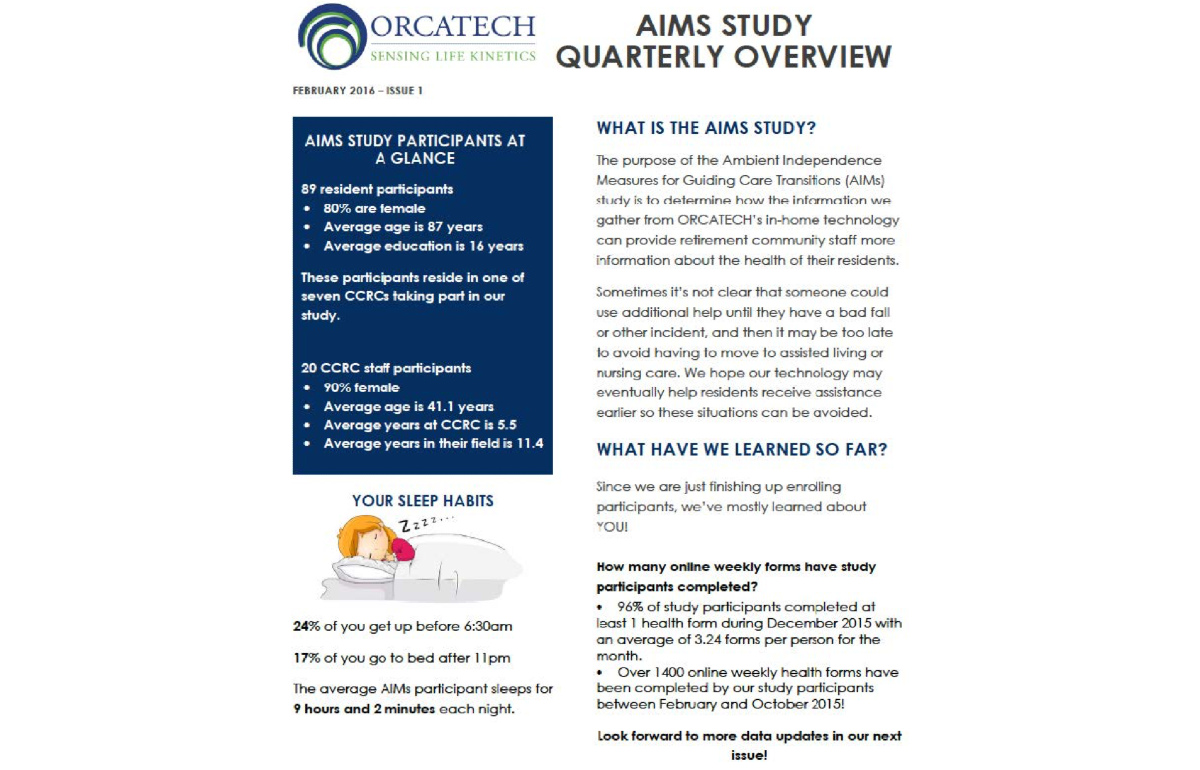
Sample page from the quarterly newsletter mailed to Ambient Independence Measures for Guiding Care Transitions (AIMS) study participants and staff. Study enrollment at the time of this newsletter was 89 residents and 20 staff.

To maintain engagement with the project, we mailed quarterly newsletters to residents and staff participants (Figure 3). Newsletters contained study updates regarding recruitment and participation, illustrative dashboard screenshots and data summaries, and *fun facts* based on data collected. For example, one newsletter presented data summarizing the sleep habits of participating residents. Newsletters to staff were accompanied by nominal gift cards to express appreciation.

### Data Collection and Analysis

For the duration of the study, dashboard access metrics were tracked, including the number of log-ins, average time spent on each dashboard page, and number of page views by each staff member.

Staff were emailed monthly surveys querying their use of the AIMS dashboard. They were asked whether they had looked at the dashboard in the last month and whether the dashboard was used in discussions about any residents. If they reported not having used the dashboard, they were asked to provide a reason. All surveys ended with an open request for feedback about the web-based activity dashboard. Midway through the study, in an effort to reengage, staff participants were sent a new survey with sample data illustrating acute and subtle changes in the behavior of one study resident. The staff responded to questions regarding the management of alerts and subsequent actions. For example, they were asked to identify preferred methods for the transmission of event reports, whether by dashboard alerts, emails, or other formats. Possible time frames for alerts, follow-up actions, and data interpretations were also probed.

At the end of the 36-month data collection period of the study, interviews with staff participants were held at 2 facilities jointly by 2 authors (KW and J Kaye) to discuss actual dashboard use among staff, study-related workload, and any other factors related to their use of the dashboard over the course of the study. The interviews were open ended and intended to elucidate barriers and opportunities for improving staff engagement in future studies (Multimedia Appendix 1).

Descriptive data for participating residents were collected at baseline. The rest of the data presented here were collected via web-based surveys and in-person interviews. Owing to small numbers, quantitative analyses of staff responses were not deemed appropriate.

### Human Subjects’ Protections

The protocol was approved by the Oregon Health & Science University Institutional Review Board (IRB#9944). Written informed consent was obtained from all participants before their inclusion in the study. The older adult residents did not receive compensation for study participation; the staff received nominal gift cards with newsletter mailings.

## Results

### Participants

A total of 95 older adult residents from 7 residential facilities in the Portland, Oregon, metro area were recruited and enrolled into the AIMS cohort (Table 2). They were 80% (76/95) female, with a mean age of 86.4 years (range 70-105 years) and Mini-Mental State Examination score of 28.7 (range 21-30). Overall, 10 participants had a Clinical Dementia Rating score of 0.5, consistent with mild cognitive impairment. Between enrollment and the data collection period, 4 participants withdrew; of the remaining 91 participants, 44 were assigned to the *viewable data* group and 47 to the *nonviewable data* group.

During data collection from December 2014 to December 2017, 6% (6/95) AIMS participants made a permanent move from independent living to assisted living or to a health care center. In addition, 32% (30/95) participants answered “yes” at least once to the weekly question regarding new care provision. The most commonly reported forms of assistance were medication management (n=22) and help with bathing, dressing, and grooming (n=19). Most of the additional assistance was provided by facility staff (n=20), whereas a minority received assistance from family or privately hired caregivers.

**Table 2:** Baseline demographic and clinical characteristics (N=95).

Variable	Value
Age (years), mean (SD)	86.4 (7.4)
Female, n (%)	76 (80)
Education (years), mean (SD)	15.9 (2.4)
Mini-Mental State Examination, mean (SD)	28.7 (1.6)
Cognitively impaired, n (%)	10 (11)
Geriatric Depression Scale, mean (SD)	1.1 (1.9)
Functional Assessment Questionnaire, mean (SD)	1.0 (3.3)
Cumulative Illness Rating Scale, mean (SD)	20.6 (2.6)

### Staff Engagement

During the 3 years of study, 11 of the 25 consented staff members logged in at least once to the activity dashboard (Table 3). All facilities were represented by at least one staff member. The number of unique log-ins to the dashboard per facility ranged from 1 to 9. Staff *page views*, that is, the number of pages of data looked at per staff member, ranged from 4 to 211 over the duration of data collection. It is clear that some staff were more engaged than others; at facility #7, 1 of 5 participating staff members logged in during the course of the study, but that staff member had 211 page views. In the 4 facilities where more than one staff member viewed the dashboard, there was consistent overlap in the residents viewed. For example, in one facility, 2 staff members viewed the same 3 residents’ data. Some residents merited multiple page views. Of the 6 residents who actually transitioned to a higher level of care during the study period, 4 had been randomly assigned to the group where monitored data were shared with staff; only 2 of these transitioned residents’ data were viewed by the staff before their transitions.

Monthly surveys were discontinued because of a low response rate. Across the 5 monthly email surveys, a total of 81 invitations to respond were sent to the staff. A total of 25 completed surveys were returned. Of the 25 surveys, in 12 instances, staff members indicated that they had looked at the web-based AIMS dashboard in the past month. Survey responses of those who had not used the dashboard in the past month (n=13) indicated that they forgot to use the dashboard (7/13, 54%), they did not need to use it because of their role in the organization (4/13, 30%), or they were unable to get onto the dashboard system because they forgot their password (2/13, 15%). Of the 12 instances where the staff indicated they had looked at the web-based AIMS dashboard, none had used the information from the dashboard as a part of a discussion about a resident they viewed.

Of the 25 eligible staff participants, 5 responded to the midstudy survey, which included sample data designed to reengage staff participants, emailed in January 2017. They generally endorsed a preference for controlling the frequency of initial alerts and follow-up reminders. Although 4 of the 5 respondents judged alerts to acute changes to be useful, only 2 respondents felt the same for alerts regarding subtle changes or trends. In open text, they explained that subtle changes in behavior were not likely to affect the overall function or well-being and were not *acute enough* to warrant their involvement. One respondent elaborated:

There is a fine line between monitoring someone’s independent lives and knowing when to interfere for safety reasons...It is hard to know when to involve a care team without being too Orwellian. I would likely wait a month and then have a bit more data to take to a team meeting to assess the subtle changes collectively.

At the end of the data collection period, 6 staff participants at 2 facilities were interviewed by 2 authors (KW and J Kaye). Interviews ranged from 1 to 1.5 hours. Feedback regarding barriers to the use of the AIMS dashboard and the data presented fell into 2 general categories.

**Table 3:** Staff engagement with the data dashboard.

Site	Number of participants consented	Dashboard use		
		Total number of participants logged	Total number of unique log-ins	Total page views
1	5	2	3	121
2	3	2	6	57
3	2	2	2	51
4	3	2	9	88
5	4	1	1	17
6	3	1	2	40
7	5	1	4	211

### Practical Issues and Barriers

Technical difficulties with the use of dashboard at the beginning of the study proved to be dissuasive for some staff members:

We had difficulty logging into the system in the beginning.

The infrequency of use exacerbated the log-in challenges because of continued unfamiliarity with the system. Furthermore, multiple staff noted that once they did access the site, the residents of interest to them in terms of changing care needs were not always study participants (because a minority of the residents they were overseeing were in the study at their site), making it less likely that they would re-enter the site. At the same time, the staff felt inundated by data in general and lacked the time to adequately review and interpret dashboard metrics. More than one staff commented that the study required a designated staff member to monitor data. Alternatively, one staff member suggested that if alerts were triaged to appropriate staff, they would all receive fewer irrelevant emails and alerts. Another staff member added that it would be critical for the system’s effectiveness that staff only receive alerts relevant to their position, “then if you get an alert you know it was meant for you.” The frequency of alerts and potential false alarms were naturally of concern in relation to time management for already overextended care providers.

Other feedback related to specific behaviors was monitored by the AIMS project. Despite initial enthusiasm about the areas of interest (eg, sleep, medication adherence, socialization), the staff subsequently recognized additional behaviors as more relevant to their decision making, such as disruptive behaviors or missing meals or appointments. At the same time, others appreciated receiving *real-time data* on metrics such as weight and sleep duration. They did acknowledge that although the residents were excited to be part of a research program, the staff felt they needed more experience with possible outcomes to see the benefits of behavior monitoring. In general, the staff struggled with the challenge of responding to acute events versus detecting trends and patterns of behavioral decline and determining how to integrate such monitoring into their daily schedules. Ultimately, some saw these data as potentially helpful in developing a model for transition, allowing them to be more proactive and less reactive.

### Professional Bias and Ethical Concerns

One interviewee acknowledged that she had biases from the beginning, in that her training as a counselor led her to be more intuitive than data driven in her decision making. Others noted their strong inclination to use the data as objective support for their own subjective perceptions of care needs.

A second concern was related to the inherent conflict between resident autonomy and safety. Multiple staff members voiced this sentiment, citing the necessary compromise between letting a resident “do what they want” even if that were to include risky behavior. However, the installation of monitoring technology raised the issue of risk management for some, in that knowledge of potentially unsafe behavior would require a decision regarding the appropriate staff response. Patient autonomy and privacy were referenced in a question posed by a director of nursing services: “How paternalistic do you want your environment to be?” An intrinsic tension between residents’ desire for control and their general willingness to share monitoring data was reflected in staff efforts to provide optimal care while respecting self-determination.

## Discussion

### Principal Findings

We report the results of developing and implementing an automated, continuous data monitoring platform that presented CCRC residents’ daily activity data on a regular basis to professional staff charged with decisions about care transitions. Our goal was to assess whether these activity metrics meaningfully contributed to this decision-making process and to test their contribution by examining in a randomized controlled trial framework whether those metrics might inform decisions regarding transitions to higher levels of care by providing early and actionable data on changes in behavior and activity.

During the 3 years of study monitoring, only 6 participants transitioned to a higher level of residential care. This number was lower than anticipated and may reflect a growing trend toward engaging additional in-home assistance instead. A total of 30 participants reported needing new in-home assistance during the study, ranging from medication management to assistance with bathing. The low rate of transitions may have contributed to the underutilization of the monitoring data dashboard by facility care staff.

Although initial acceptance of the project was enthusiastic, with the staff in 7 facilities committing to regular utilization of the data dashboard, this enthusiasm failed to carry over into implementation. The staff used the dashboard sporadically, and those who did identified several limitations to use, ranging from technology challenges to ethical concerns.

### Comparisons With Previous Work

Previous work on the adoption of technology by health care professionals has used various iterations of the Technology Acceptance Model (TAM) [23]. This model postulates that the intention to use technology is predicated on attitudes that are mediated by the perceived attributes of the technology. The two most important attributes in explaining acceptance and use of technology have been proposed to be perceived usefulness and perceived ease of use [24]. Modified technology acceptance models have added subjective norms and facilitator conditions as determinants of intent to use [25].

Although all models have been shown to have some explanatory power, research applications have typically incorporated *behavioral intention to use* rather than actual use. Few studies include measures of actual technology use, relying instead on measures of behavioral intent. Our experience in this project suggests that despite perceived usefulness at baseline and attempts to accommodate users’ needs to achieve perceived ease of use, actual technology use can still be lower than predicted by indicators of intent to use.

TAMs have been applied to the identification of barriers to adoption by health care providers. Organizational factors such as administrative leadership and support, including additional time allotment and clear incentives, adequate resources for training and ongoing technical support, and organizational planning for implementation have been cited as important barriers [26-29]. Technical impediments include malfunctioning or unreliable equipment and devices and lack of coordination or complementarity with existing procedures. Failure to include potential end users in the design and planning of technology applications has also been cited as an impediment to adoption [27,30]. In addition, the ability of users to exert control over the technology’s behavior has been cited as an important motivator in the adoption of a new technology [31]. Sabrowski and Kollak [32] describe the *domestication* of technology as a process whereby the system or device is integrated and adapted to the user’s needs and environment. They postulate that until care professionals view a technology as integral to an improvement in the delivery of care, they will be resistant to adoption. Finally, human factors connected to attitudes and previous experiences with technology can have enormous influence. Lack of knowledge or familiarity with a device or system can diminish both perceived usefulness and ease of use. Furthermore, for health care providers seeking to maximize the quality of life for a medically fragile population, concerns about loss of human contact can foster negative attitudes toward technology. Savenstedt et al [33] identified themes elicited from interviews with professional caregivers of older adults. Technology applications were seen as both an aid and a threat to not only humane care but also to their roles as caregivers. They cited the loss of immediate contact and involvement with their care recipients as a potential consequence of technology applications. The authors suggest that these inherent conflicts foster resistance to change despite outward acceptance.

### Limitations and Lessons Learned

We found sporadic adoption of a new monitoring technology by professional staff. Despite initial enthusiasm and ongoing efforts to engage the participating staff in 7 residential care communities, the goal of this study, that is, to analyze the impact of technology-based data on decision making around transitions in care, was not achieved. Previous research has described organizational, personal, and technological characteristics and contexts that may facilitate or impede the adoption of health technologies [29,34-37]. Feedback from our staff participants was consistent with these barriers to technology use.

### Organizational Barriers to Use

Organizational factors such as clearly communicated expectations and possible study outcomes, continuous monitoring of technical support needs of the staff, and recognition of time commitments may have been inadequately addressed. A consistent recommendation has been the early inclusion of end users in design and implementation. Although our initial focus groups elicited some preferences and priorities, a longer run-in iterative process might have reduced the gap between our efforts at participatory design and the reality of the final implementation. Although some staff members recommend identifying a *champion* or *super-user* at each facility to provide onsite, continuous support and motivation to engage with the platform [37], we found few staff members who self-identified as such. Other organizational factors, although beyond the scope of this project, should be considered moving forward. A unified commitment to the implementation of new technologies by the administration, staff, and residents is required and must include the provision of adequate time for staff education and training, recognition of professional autonomy, and ongoing identification of potential barriers.

### Personal and Professional Barriers to Use

Personal and professional traits related to technology adoption include experience with technology, peer attitudes, staff engagement, and professional satisfaction [29,34]. Although initial training was provided to all staff in dashboard use and features, additional active ongoing technical support might have increased engagement. Perceived usefulness may have been diminished because of lack of concordance between resident research participants and particular residents of interest (who were not monitored in the study) to the transition teams.

The staff expressed ethical concerns related to the quality of care and privacy. Previously, unexamined conflicted attitudes toward monitoring technology surfaced only after actual engagement with the system. A more detailed discussion of the implications and possible outcomes of staff participation in a continuous monitoring study before implementation might have mitigated ethical apprehensions. An increase in staff workload or change in procedure, without adequate motivation and explanation, cannot be expected to be enthusiastically adopted. In the future, identification of intrinsic and extrinsic motivators, adequate and sustained training, and a realistic understanding of the goals of the study must be an integral part of research in technology adoption.

### Technical Barriers to Use

Finally, issues with the technology itself may have presented barriers to implementation. Technical issues such as failed passwords and initial platform malfunctions, while infrequent, led to some early negative interactions, which proved to be difficult to overcome. In addition to the initial discussion of needs with end users, further refinement of the platform might have enhanced sustained participation. However, the refinement of protocols must be balanced with the time and resources needed to implement a program, acknowledging that staff turnover can attenuate involvement over time.

The original premise of our intervention was that a less obtrusive, information-on-demand approach would be least disruptive to workflows. However, at least initially, sending notifications of changes in resident behavior rather than relying on the staff to engage and retrieve data from a novel and unfamiliar source might have increased their understanding of the utility of the technology and its relevance to their daily practice. Providing actionable, customized information on residents at risk would demonstrate the potential benefits of continuous monitoring over standard procedures regarding transitions in care.

### Conclusions

The limitations of previous work describing the intent to use technology without the inclusion of actual use as the final outcome are demonstrated by our findings. Initial enthusiasm and support for in-home, continuous monitoring of activity and behavior was established among the staff of 7 continuing care residential communities. Nevertheless, multiple factors, whether technical, personal, or institutional, intervened between intent and use. Future research examining technology adoption cannot ignore this crucial outcome measure if widespread acceptance and implementation of health care technologies are to be advanced. Finally, and perhaps most importantly, future work should examine whether a culture change toward proactive intervention to prevent or safely delay unwanted care transitions, rather than using technology for emergency response and acute situational management, will achieve wider use of technologies across residential care communities and related settings.

## Appendix

Multimedia Appendix 1 End of study discussion guide.

## Abbreviations

AIMS: Ambient Independence Measures for Guiding Care Transitions
CCRC: continuing care retirement community
TAM: Technology Acceptance Model
ORCATECH: Oregon Center for Aging & Technology
