# Supplementary material for: Application of In-Home Monitoring Data to Transition Decisions in Continuing Care Retirement Communities: Usability Study
Source: J Med Internet Res. 2021 Jan 13;23(1):e18806. doi: 10.2196/18806 (PMC7840292; doi:10.2196/18806)
Supplement: Multimedia Appendix 1 [file jmir_v23i1e18806_app1.docx]

**AIMS End of Study Discussion Guide**

*Actual Dashboard Use*

Did you have a chance to use the dashboard?

How often?

How many residents?

Was data from the dashboard ever discussed during staff meetings about residents?

Was data ever shared with residents’ families? MD’s?

Were the residents who were monitored not the ones of interest to the transition team?

Tell us about the relative importance of alerts for acute vs. subtle or chronic changes.

*Study related work load*

Did the fact of monitoring put pressure on the staff to pay more attention to those residents?

Did the dashboard create extra work for the staff in any way?

*Dashboard Issues*

Was the timing of alerts appropriate?

What would your preferences have been for receiving alerts?
 Weekly?

As they occur?

How user friendly was the dashboard?

What problems did you have with it?

What could it have done better or differently?
